# (Biphenyl-2,2′-di­yl)di-*tert*-butyl­phos­phonium trifluoro­methane­sulfonate

**DOI:** 10.1107/S1600536812049045

**Published:** 2012-12-05

**Authors:** Alfred Muller, Cedric W. Holzapfel

**Affiliations:** a Department of Chemistry, University of Johannesburg (APK Campus), PO Box 524, Auckland Park, Johannesburg, 2006, South Africa

## Abstract

To aid in the elucidation of catalytic reaction mechanism of palladacycles, we found that reaction of trifluoro­methane­sulfonic acid with a phosphapalladacycle resulted in elimination of the palladium and formation of the title phospholium salt, C_20_H_26_P^+^·CF_3_SO_3_^−^. Selected geometrical parameters include P—biphenyl (av.) = 1.801 (3) Å and P—*t*-Bu (av.) = 1.858 (3) Å, and significant distortion of the tetra­hedral P-atom environment with biphen­yl—P—biphenyl = 93.93 (13)° and *t*-Bu—P—*t*-Bu = 118.82 (14)°. In the crystal, weak C—H⋯O inter­actions lead to channels along the *c* axis that are occupied by CF_3_SO_3_^−^ anions.

## Related literature

For background to catalytic studies on palladacycles, see: Herrman *et al.* (2003 ▶); Beletskaya & Cheprakov (2004 ▶); Omondi *et al.* (2011 ▶); Williams *et al.* (2008 ▶); d’Orlye & Jutland (2005 ▶). For a description of the Cambridge Structural Database, see: Allen (2002 ▶).
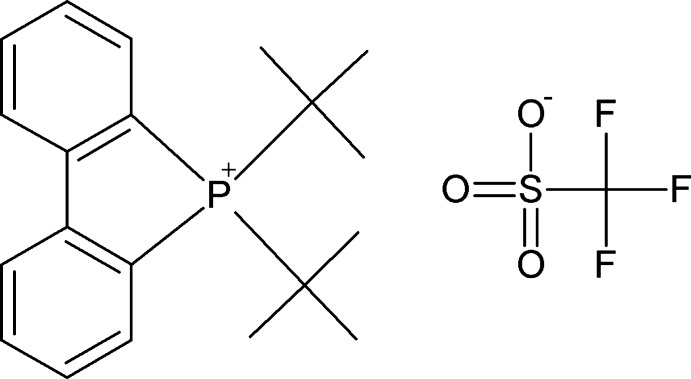


## Experimental

### 

#### Crystal data

C_20_H_26_P^+^·CF_3_O_3_S^−^
*M* *_r_* = 446.45
Tetragonal, 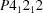

*a* = 12.1339 (10) Å
*c* = 30.057 (2) Å
*V* = 4425.4 (6) Å^3^
*Z* = 8
Mo *K*α radiation
μ = 0.26 mm^−1^
*T* = 293 K
0.4 × 0.26 × 0.2 mm

#### Data collection

Bruker SMART 1K CCD diffractometer
Absorption correction: multi-scan (*SADABS*; Bruker, 2008 ▶) *T*_min_ = 0.902, *T*_max_ = 0.949
25275 measured reflections
5502 independent reflections
3241 reflections with *I* > 2σ(*I*)
*R*_int_ = 0.101

#### Refinement

*R*[*F* ^2^ > 2σ(*F* ^2^)] = 0.054
*wR*(*F* ^2^) = 0.117
*S* = 0.99
5502 reflections
268 parameters
H-atom parameters constrained
Δρ_max_ = 0.17 e Å^−3^
Δρ_min_ = −0.25 e Å^−3^
Absolute structure: Flack (1983 ▶), 2283 Friedel pairs
Flack parameter: 0.05 (11)

### 

Data collection: *SMART-NT* (Bruker, 1998 ▶); cell refinement: *SAINT-Plus* (Bruker, 2008 ▶); data reduction: *SAINT-Plus* and *XPREP* (Bruker, 2008 ▶); program(s) used to solve structure: *SIR97* (Altomare *et al.*, 1999 ▶); program(s) used to refine structure: *SHELXL97* (Sheldrick, 2008 ▶); molecular graphics: *DIAMOND* (Brandenburg & Putz, 2005 ▶); software used to prepare material for publication: *WinGX* (Farrugia, 2012 ▶).

## Supplementary Material

Crystal structure: contains datablock(s) global, I. DOI: 10.1107/S1600536812049045/aa2079sup1.cif

Structure factors: contains datablock(s) I. DOI: 10.1107/S1600536812049045/aa2079Isup2.hkl

Additional supplementary materials:  crystallographic information; 3D view; checkCIF report

## Figures and Tables

**Table 1:** Hydrogen-bond geometry (Å, °)

*D*—H⋯*A*	*D*—H	H⋯*A*	*D*⋯*A*	*D*—H⋯*A*
C2—H2⋯O1^i^	0.93	2.49	3.381 (4)	161
C19—H19*A*⋯O2^ii^	0.96	2.52	3.458 (4)	165
C11—H11⋯O3^iii^	0.93	2.70	3.601 (4)	162
C15—H15*B*⋯O3^iii^	0.96	2.69	3.470 (4)	138

Symmetry codes: (i) 
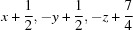
; (ii) 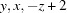; (iii) 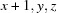.

**Fig. 1.**
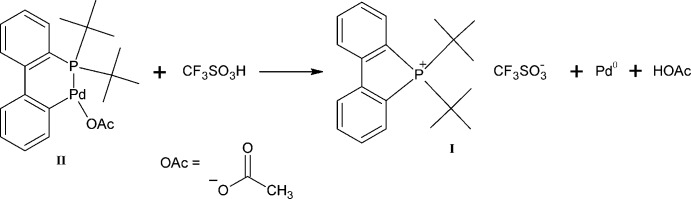
Proposed reaction scheme of the elimination of the palladium and formation of the title phospholium compound.

**Fig. 2.**
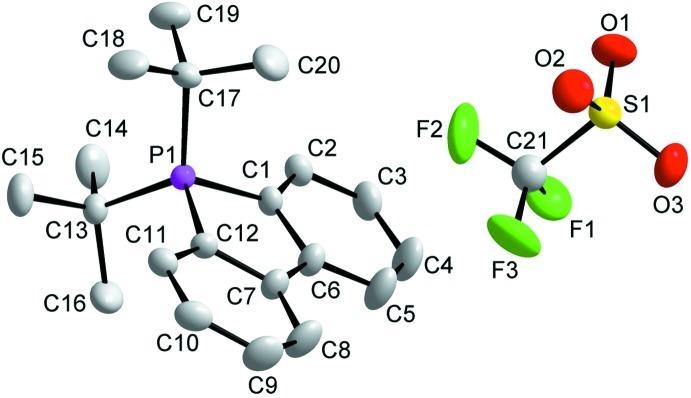
View of title compound showing displacement ellipsoids (drawn at a 30% probability level) and numbering scheme. Hydrogen atoms have been omitted for clarity.

## supplementary crystallographic information

### Comment

The introduction of a cyclopalladated compound as a robust catalyst (Herrman *et al.*, 2003) for Heck and cross-coupling reactions resulted in the design of structurally related palladacycles, phosphapalladacycles in particular (Beletskaya & Cheprakov, 2004). However, growing evidence suggests that palladacycles are disassembled during the pre-activation stage to yield low ligated Pd^0^ species as the actual catalyst (d'Orlye & Jutland, 2005). This is further exemplified by our finding that the use of palladacycle (II in Fig. 1), an effective amination catalyst (Beletskaya & Cheprakov, 2004), as a source of palladium together with triphenylphosphine and a strong acid provided an extremely active catalytic system for the hydromethoxylation of alkenes (Omondi *et al.*, 2011 and Williams *et al.*, 2008). In the reaction medium compound II (Fig. 1) was rapidly converted into tetrakistriphenylphosphinepalladium(0) which acted as the actual catalyst. The facile formation of Pd^0^ resulted from acid catalyzed elimination from the palladacycle. This treatment of II with ten equivalents of trifluoromethanesulfonic acid at room temperature resulted in the formation of colloidal palladium and the title compound (I in Fig. 1), the structure of which was confirmed by single-crystal X-ray crystallography.

The title compound I (Fig. 2 and Scheme 1) is a salt consisting of phospholium cations and trifluoromethanesulfonate anions. All ions lie on general positions in the unit cell with no discernible differences in the bond distances of the coordination polyhedron of the phosphorus environment. The bond angles at the phosphorus center shows significant deviations from the expected 109.5° for the tetrahedral shape with biphenyl—P—biphenyl = 93.93 (13)° and *t*-Bu—P—*t*-Bu = 118.82 (14)°. These deviations can be ascribed to the somewhat strained 5-membered cyclisation of the dibenzo fragment to form the phospholium ring. This pinching effect in turn allows for more space for the bulky tertiary butyl groups positioned above and below the plane formed by the tricyclic phospholium conjugate and hence the observed *t*-Bu—P—*t*-Bu angle. The tricyclic phospholium conjugate marginally deviates from planarity (C1—C6—C7—C12 = 1.9 (4)°), and would have been the primary route to alleviate stress from the pinching effect. Data extracted from the Cambridge Structural Database (Allen, 2002) for the torsion angle between the planes shows a mean value of 1.72° (126 observations). The general trend seems to be that substituents opposite the phospholium cycle forces it to be planar. The preferred orientation of the tertiary butyls are due to several weak C—H···O interactions observed between ions (see Table 1).

### Experimental

Trifluorometanesulfonic acid (150 mg, 1 mmol) in 5 ml me thanol was added dropwise to a stirred solution of (acetato-κ^2^O,O')[2'-(di-*tert*- butylphosphanyl)-1,1'-biphenyl-κ^2^P,C^2^]palladium (462 mg, 1 mmol) in dichloromethane (30 ml) at room temperature under argon. The solution changed from colourless to deep purple and then to dark brown over a period of 10 min. After several hours at room temperature a fine precipitate of palladium black started to form. After 24 h the reaction mixture was filtered through celite to remove the palladium. The solvent was removed *in vacuo* and the residue distributed between water (15 ml) and ether (15 ml). The aqueous phase was extracted with dichloromethane (3 *x* 30 ml). The combined extract furnished crystalline (413 mg, 82%) on removal of the solvent *in vacuo*. Good crystals (mp. 157 – 159 °C) was obtained by diffusion of the vapours of ether into a solution in dichloromethane. Analytical data: ^1^H-NMR: δ 1.45 (18*H*, d, *J* = 17 Hz), 7.68 (2*H*, dt, *J* = 2.5 and 7.5 Hz), 7.85 (2*H*, t, *J* = 7.8 Hz), 7.99 (2*H*, t, *J* = 7.8 Hz), and 8.64 (2*H*, dd, *J* = 2.5 and 7.8 Hz) ^13^C{H}-NMR: δ 26.74 (*s*), 36.42 (d, *J* = 42 Hz), 118.72 (d, *J* = 101 Hz), 123.58 (d, *J* = 12.3 Hz), 130.90 (d, *J* = 14.0 Hz), 131.90 (d, *J* = 14.0 Hz), 131.67 (d, *J* = 11.4 Hz), 135.95 (d, *J* = 2.7 Hz), 144.97 (d, *J* = 17.4 Hz) ^31^P-NMR: δ 51.24

### Refinement

All hydrogen atoms for methyl and aromatic H atoms were positioned in geometrically idealized positions with C—H = 0.96 Å and 0.93 Å respectively. Aromatic hydrogen atoms were allowed to ride on their parent atoms with *U*_iso_(H) = 1.2*U*_eq_, and for methyl hydrogen atoms *U*_iso_(H) = 1.5*U*_eq_ was utilized. The initial positions of methyl hydrogen atoms were located from a Fourier difference map and refined as fixed rotor. The Flack parameter refined to 0.05 (11).

### Crystal data

**Table:**

C_20_H_26_P^+^·CF_3_O_3_S^−^	*D*_x_ = 1.34 Mg m^−^^3^
*M**_r_* = 446.45	Mo *K*α radiation, λ = 0.71073 Å
Tetragonal, *P*4_1_2_1_2	Cell parameters from 3572 reflections
Hall symbol: P 4abw 2nw	θ = 2.5–21.9°
*a* = 12.1339 (10) Å	µ = 0.26 mm^−^^1^
*c* = 30.057 (2) Å	*T* = 293 K
*V* = 4425.4 (6) Å^3^	Prism, yellow
*Z* = 8	0.4 × 0.26 × 0.2 mm
*F*(000) = 1872	

### Data collection

**Table:**

Bruker SMART 1K CCD diffractometer	3241 reflections with *I* > 2σ(*I*)
Graphite monochromator	*R*_int_ = 0.101
π scans	θ_max_ = 28.4°, θ_min_ = 1.8°
Absorption correction: multi-scan (*SADABS*; Bruker, 2008)	*h* = −13→16
*T*_min_ = 0.902, *T*_max_ = 0.949	*k* = −11→16
25275 measured reflections	*l* = −39→28
5502 independent reflections	

### Refinement

**Table:**

Refinement on *F*^2^	Secondary atom site location: difference Fourier map
Least-squares matrix: full	Hydrogen site location: inferred from neighbouring sites
*R*[*F*^2^ > 2σ(*F*^2^)] = 0.054	H-atom parameters constrained
*wR*(*F*^2^) = 0.117	*w* = 1/[σ^2^(*F*_o_^2^) + (0.0519*P*)^2^] where *P* = (*F*_o_^2^ + 2*F*_c_^2^)/3
*S* = 0.99	(Δ/σ)_max_ = 0.001
5502 reflections	Δρ_max_ = 0.17 e Å^−^^3^
268 parameters	Δρ_min_ = −0.25 e Å^−^^3^
0 restraints	Absolute structure: Flack (1983), 2283 Friedel pairs
Primary atom site location: structure-invariant direct methods	Flack parameter: 0.05 (11)

### Special details

**Table:**

Experimental. The intensity data was collected on a Bruker *SMART* 1 K CCD diffractometer using an exposure time of 20 s/frame. A total of 984 frames were collected with a frame width of 0.3° covering up to θ = 28.37° with 99.3% completeness accomplished.
Geometry. All e.s.d.'s (except the e.s.d. in the dihedral angle between two l.s. planes) are estimated using the full covariance matrix. The cell e.s.d.'s are taken into account individually in the estimation of e.s.d.'s in distances, angles and torsion angles; correlations between e.s.d.'s in cell parameters are only used when they are defined by crystal symmetry. An approximate (isotropic) treatment of cell e.s.d.'s is used for estimating e.s.d.'s involving l.s. planes.
Refinement. Refinement of *F*^2^ against ALL reflections. The weighted *R*-factor *wR* and goodness of fit *S* are based on *F*^2^, conventional *R*-factors *R* are based on *F*, with *F* set to zero for negative *F*^2^. The threshold expression of *F*^2^ > σ(*F*^2^) is used only for calculating *R*-factors(gt) *etc*. and is not relevant to the choice of reflections for refinement. *R*-factors based on *F*^2^ are statistically about twice as large as those based on *F*, and *R*- factors based on ALL data will be even larger.

### Fractional atomic coordinates and isotropic or equivalent isotropic displacement parameters (Å^2^)

**Table:**

	*x*	*y*	*z*	*U*_iso_*/*U*_eq_	
P1	0.60082 (6)	0.44749 (6)	0.87672 (2)	0.03329 (18)	
C1	0.4783 (2)	0.4567 (2)	0.84292 (9)	0.0370 (7)	
C2	0.4328 (2)	0.3813 (3)	0.81337 (9)	0.0454 (8)	
H2	0.4651	0.3126	0.8092	0.054*	
C3	0.3384 (3)	0.4104 (3)	0.79028 (11)	0.0576 (9)	
H3	0.3082	0.3616	0.7698	0.069*	
C4	0.2892 (3)	0.5108 (3)	0.79748 (13)	0.0744 (12)	
H4	0.2255	0.5289	0.7819	0.089*	
C5	0.3324 (3)	0.5852 (3)	0.82750 (13)	0.0746 (12)	
H5	0.2979	0.6525	0.8322	0.09*	
C6	0.4280 (2)	0.5587 (3)	0.85057 (10)	0.0472 (8)	
C7	0.4846 (2)	0.6282 (2)	0.88434 (10)	0.0450 (7)	
C8	0.4517 (3)	0.7307 (3)	0.89949 (13)	0.0692 (11)	
H8	0.3888	0.7639	0.888	0.083*	
C9	0.5129 (4)	0.7833 (3)	0.93185 (12)	0.0687 (11)	
H9	0.4896	0.8516	0.9424	0.082*	
C10	0.6074 (3)	0.7372 (3)	0.94885 (10)	0.0536 (9)	
H10	0.6475	0.774	0.9706	0.064*	
C11	0.6428 (3)	0.6358 (3)	0.93346 (9)	0.0427 (8)	
H11	0.7076	0.6048	0.9443	0.051*	
C12	0.5808 (2)	0.5806 (2)	0.90172 (9)	0.0359 (7)	
C13	0.7242 (2)	0.4477 (3)	0.83999 (9)	0.0402 (7)	
C14	0.7210 (3)	0.3492 (3)	0.80819 (11)	0.0714 (12)	
H14A	0.7818	0.3538	0.7878	0.107*	
H14B	0.653	0.3501	0.7918	0.107*	
H14C	0.7262	0.2821	0.8249	0.107*	
C15	0.8297 (3)	0.4475 (3)	0.86695 (11)	0.0643 (10)	
H15A	0.8327	0.382	0.8848	0.096*	
H15B	0.8314	0.5112	0.8859	0.096*	
H15C	0.8919	0.4491	0.8472	0.096*	
C16	0.7161 (3)	0.5544 (3)	0.81278 (12)	0.0724 (11)	
H16A	0.7235	0.6167	0.8322	0.109*	
H16B	0.6458	0.5573	0.7981	0.109*	
H16C	0.7738	0.5558	0.7909	0.109*	
C17	0.5876 (3)	0.3380 (2)	0.91950 (10)	0.0441 (8)	
C18	0.6709 (3)	0.3567 (3)	0.95735 (11)	0.0679 (11)	
H18A	0.6629	0.2996	0.9792	0.102*	
H18B	0.6574	0.4271	0.9709	0.102*	
H18C	0.7444	0.3552	0.9455	0.102*	
C19	0.6031 (3)	0.2239 (3)	0.89885 (11)	0.0651 (10)	
H19A	0.5876	0.1684	0.9207	0.098*	
H19B	0.6777	0.2162	0.8887	0.098*	
H19C	0.5536	0.2156	0.8742	0.098*	
C20	0.4700 (3)	0.3478 (3)	0.93813 (13)	0.0797 (13)	
H20A	0.4177	0.3361	0.9146	0.12*	
H20B	0.4596	0.42	0.9505	0.12*	
H20C	0.4592	0.2934	0.9609	0.12*	
C21	0.1201 (3)	0.5421 (4)	0.93747 (13)	0.0681 (11)	
S1	0.01008 (7)	0.49559 (7)	0.97325 (2)	0.0494 (2)	
O1	−0.0109 (2)	0.3861 (2)	0.95856 (8)	0.0773 (8)	
O2	0.0560 (2)	0.5059 (2)	1.01687 (8)	0.0780 (8)	
O3	−0.0772 (2)	0.5722 (2)	0.96379 (8)	0.0762 (8)	
F1	0.0927 (2)	0.5358 (2)	0.89479 (7)	0.0956 (8)	
F2	0.2088 (2)	0.4800 (3)	0.94266 (10)	0.1323 (12)	
F3	0.1493 (2)	0.6442 (2)	0.94525 (9)	0.1194 (10)	

### Atomic displacement parameters (Å^2^)

**Table:**

	*U*^11^	*U*^22^	*U*^33^	*U*^12^	*U*^13^	*U*^23^
P1	0.0327 (4)	0.0315 (4)	0.0357 (4)	0.0012 (3)	−0.0020 (3)	−0.0017 (3)
C1	0.0316 (17)	0.0394 (17)	0.0402 (17)	−0.0015 (13)	−0.0040 (12)	−0.0074 (13)
C2	0.0408 (19)	0.046 (2)	0.0499 (18)	−0.0048 (14)	−0.0006 (14)	−0.0101 (14)
C3	0.043 (2)	0.073 (3)	0.057 (2)	−0.0074 (19)	−0.0131 (16)	−0.0140 (19)
C4	0.054 (2)	0.082 (3)	0.087 (3)	0.016 (2)	−0.036 (2)	−0.019 (2)
C5	0.059 (3)	0.068 (3)	0.097 (3)	0.025 (2)	−0.031 (2)	−0.023 (2)
C6	0.0405 (19)	0.0486 (19)	0.0527 (19)	0.0085 (15)	−0.0091 (14)	−0.0083 (16)
C7	0.0429 (19)	0.0394 (18)	0.0527 (19)	0.0092 (14)	−0.0024 (14)	−0.0075 (14)
C8	0.076 (3)	0.052 (2)	0.080 (3)	0.028 (2)	−0.016 (2)	−0.0170 (19)
C9	0.089 (3)	0.043 (2)	0.074 (3)	0.015 (2)	−0.001 (2)	−0.0187 (18)
C10	0.074 (3)	0.044 (2)	0.0424 (19)	−0.0093 (19)	0.0009 (18)	−0.0068 (15)
C11	0.0457 (19)	0.0417 (19)	0.0406 (18)	−0.0064 (15)	−0.0008 (14)	−0.0039 (14)
C12	0.0392 (18)	0.0305 (17)	0.0379 (16)	−0.0011 (12)	0.0000 (13)	−0.0047 (12)
C13	0.0338 (17)	0.0500 (19)	0.0366 (17)	0.0014 (14)	−0.0006 (13)	−0.0024 (15)
C14	0.062 (3)	0.091 (3)	0.061 (2)	0.004 (2)	0.0162 (19)	−0.030 (2)
C15	0.038 (2)	0.100 (3)	0.055 (2)	0.0045 (19)	−0.0035 (16)	−0.007 (2)
C16	0.059 (2)	0.084 (3)	0.075 (3)	0.001 (2)	0.0156 (19)	0.034 (2)
C17	0.048 (2)	0.0409 (19)	0.0433 (18)	−0.0060 (14)	0.0026 (15)	0.0063 (13)
C18	0.088 (3)	0.061 (2)	0.055 (2)	−0.008 (2)	−0.019 (2)	0.0175 (18)
C19	0.090 (3)	0.042 (2)	0.063 (2)	−0.0063 (19)	−0.011 (2)	0.0065 (17)
C20	0.069 (3)	0.092 (3)	0.078 (3)	−0.004 (2)	0.025 (2)	0.026 (2)
C21	0.065 (3)	0.072 (3)	0.068 (3)	−0.002 (2)	−0.002 (2)	−0.003 (2)
S1	0.0554 (5)	0.0515 (5)	0.0412 (4)	−0.0037 (4)	−0.0034 (4)	−0.0056 (4)
O1	0.103 (2)	0.0526 (16)	0.0761 (17)	−0.0131 (15)	−0.0052 (16)	−0.0034 (13)
O2	0.0889 (19)	0.102 (2)	0.0427 (14)	−0.0036 (16)	−0.0149 (12)	−0.0077 (13)
O3	0.0609 (18)	0.089 (2)	0.0783 (16)	0.0226 (14)	−0.0057 (13)	−0.0149 (15)
F1	0.109 (2)	0.125 (2)	0.0528 (13)	−0.0194 (16)	0.0148 (12)	0.0069 (13)
F2	0.0551 (16)	0.197 (4)	0.145 (2)	0.036 (2)	0.0137 (16)	−0.001 (2)
F3	0.139 (3)	0.092 (2)	0.128 (2)	−0.0655 (18)	0.0139 (19)	−0.0078 (16)

### Geometric parameters (Å, º)

**Table:**

P1—C12	1.798 (3)	C14—H14A	0.96
P1—C1	1.804 (3)	C14—H14B	0.96
P1—C17	1.856 (3)	C14—H14C	0.96
P1—C13	1.860 (3)	C15—H15A	0.96
C1—C2	1.389 (4)	C15—H15B	0.96
C1—C6	1.400 (4)	C15—H15C	0.96
C2—C3	1.384 (4)	C16—H16A	0.96
C2—H2	0.93	C16—H16B	0.96
C3—C4	1.374 (5)	C16—H16C	0.96
C3—H3	0.93	C17—C19	1.529 (4)
C4—C5	1.380 (5)	C17—C20	1.538 (5)
C4—H4	0.93	C17—C18	1.538 (4)
C5—C6	1.388 (4)	C18—H18A	0.96
C5—H5	0.93	C18—H18B	0.96
C6—C7	1.487 (4)	C18—H18C	0.96
C7—C8	1.383 (4)	C19—H19A	0.96
C7—C12	1.403 (4)	C19—H19B	0.96
C8—C9	1.380 (5)	C19—H19C	0.96
C8—H8	0.93	C20—H20A	0.96
C9—C10	1.376 (5)	C20—H20B	0.96
C9—H9	0.93	C20—H20C	0.96
C10—C11	1.382 (4)	C21—F3	1.310 (4)
C10—H10	0.93	C21—F2	1.323 (5)
C11—C12	1.387 (4)	C21—F1	1.327 (4)
C11—H11	0.93	C21—S1	1.805 (4)
C13—C15	1.515 (4)	S1—O1	1.423 (3)
C13—C14	1.531 (4)	S1—O2	1.430 (2)
C13—C16	1.534 (4)	S1—O3	1.438 (2)
			
C12—P1—C1	93.93 (13)	C13—C14—H14C	109.5
C12—P1—C17	109.98 (13)	H14A—C14—H14C	109.5
C1—P1—C17	111.29 (14)	H14B—C14—H14C	109.5
C12—P1—C13	110.82 (14)	C13—C15—H15A	109.5
C1—P1—C13	109.20 (12)	C13—C15—H15B	109.5
C17—P1—C13	118.82 (14)	H15A—C15—H15B	109.5
C2—C1—C6	120.9 (3)	C13—C15—H15C	109.5
C2—C1—P1	130.3 (2)	H15A—C15—H15C	109.5
C6—C1—P1	108.8 (2)	H15B—C15—H15C	109.5
C3—C2—C1	118.8 (3)	C13—C16—H16A	109.5
C3—C2—H2	120.6	C13—C16—H16B	109.5
C1—C2—H2	120.6	H16A—C16—H16B	109.5
C4—C3—C2	120.4 (3)	C13—C16—H16C	109.5
C4—C3—H3	119.8	H16A—C16—H16C	109.5
C2—C3—H3	119.8	H16B—C16—H16C	109.5
C3—C4—C5	121.2 (3)	C19—C17—C20	109.4 (3)
C3—C4—H4	119.4	C19—C17—C18	110.7 (3)
C5—C4—H4	119.4	C20—C17—C18	109.2 (3)
C4—C5—C6	119.6 (3)	C19—C17—P1	110.9 (2)
C4—C5—H5	120.2	C20—C17—P1	106.1 (2)
C6—C5—H5	120.2	C18—C17—P1	110.5 (2)
C5—C6—C1	119.1 (3)	C17—C18—H18A	109.5
C5—C6—C7	126.6 (3)	C17—C18—H18B	109.5
C1—C6—C7	114.3 (3)	H18A—C18—H18B	109.5
C8—C7—C12	119.1 (3)	C17—C18—H18C	109.5
C8—C7—C6	127.0 (3)	H18A—C18—H18C	109.5
C12—C7—C6	113.9 (3)	H18B—C18—H18C	109.5
C9—C8—C7	119.6 (3)	C17—C19—H19A	109.5
C9—C8—H8	120.2	C17—C19—H19B	109.5
C7—C8—H8	120.2	H19A—C19—H19B	109.5
C10—C9—C8	121.5 (3)	C17—C19—H19C	109.5
C10—C9—H9	119.3	H19A—C19—H19C	109.5
C8—C9—H9	119.3	H19B—C19—H19C	109.5
C9—C10—C11	119.8 (3)	C17—C20—H20A	109.5
C9—C10—H10	120.1	C17—C20—H20B	109.5
C11—C10—H10	120.1	H20A—C20—H20B	109.5
C10—C11—C12	119.4 (3)	C17—C20—H20C	109.5
C10—C11—H11	120.3	H20A—C20—H20C	109.5
C12—C11—H11	120.3	H20B—C20—H20C	109.5
C11—C12—C7	120.6 (3)	F3—C21—F2	107.3 (4)
C11—C12—P1	130.3 (2)	F3—C21—F1	107.1 (3)
C7—C12—P1	109.1 (2)	F2—C21—F1	106.6 (3)
C15—C13—C14	110.7 (3)	F3—C21—S1	112.9 (3)
C15—C13—C16	109.9 (3)	F2—C21—S1	110.7 (3)
C14—C13—C16	108.9 (3)	F1—C21—S1	111.9 (3)
C15—C13—P1	111.3 (2)	O1—S1—O2	115.83 (15)
C14—C13—P1	110.4 (2)	O1—S1—O3	114.24 (17)
C16—C13—P1	105.4 (2)	O2—S1—O3	114.32 (15)
C13—C14—H14A	109.5	O1—S1—C21	103.84 (18)
C13—C14—H14B	109.5	O2—S1—C21	103.35 (17)
H14A—C14—H14B	109.5	O3—S1—C21	103.01 (18)
			
C12—P1—C1—C2	−178.5 (3)	C17—P1—C12—C11	64.7 (3)
C17—P1—C1—C2	−65.2 (3)	C13—P1—C12—C11	−68.7 (3)
C13—P1—C1—C2	67.9 (3)	C1—P1—C12—C7	−0.2 (2)
C12—P1—C1—C6	1.2 (2)	C17—P1—C12—C7	−114.5 (2)
C17—P1—C1—C6	114.4 (2)	C13—P1—C12—C7	112.1 (2)
C13—P1—C1—C6	−112.5 (2)	C12—P1—C13—C15	74.3 (3)
C6—C1—C2—C3	2.3 (4)	C1—P1—C13—C15	176.4 (2)
P1—C1—C2—C3	−178.1 (2)	C17—P1—C13—C15	−54.5 (3)
C1—C2—C3—C4	−1.8 (5)	C12—P1—C13—C14	−162.4 (2)
C2—C3—C4—C5	0.4 (6)	C1—P1—C13—C14	−60.2 (3)
C3—C4—C5—C6	0.6 (6)	C17—P1—C13—C14	68.8 (3)
C4—C5—C6—C1	−0.1 (6)	C12—P1—C13—C16	−44.9 (2)
C4—C5—C6—C7	−179.1 (4)	C1—P1—C13—C16	57.2 (2)
C2—C1—C6—C5	−1.3 (5)	C17—P1—C13—C16	−173.7 (2)
P1—C1—C6—C5	179.0 (3)	C12—P1—C17—C19	179.3 (2)
C2—C1—C6—C7	177.8 (3)	C1—P1—C17—C19	76.6 (3)
P1—C1—C6—C7	−1.9 (3)	C13—P1—C17—C19	−51.5 (3)
C5—C6—C7—C8	1.2 (6)	C12—P1—C17—C20	60.7 (3)
C1—C6—C7—C8	−177.9 (3)	C1—P1—C17—C20	−42.0 (3)
C5—C6—C7—C12	−179.1 (3)	C13—P1—C17—C20	−170.1 (2)
C1—C6—C7—C12	1.9 (4)	C12—P1—C17—C18	−57.6 (3)
C12—C7—C8—C9	−1.1 (5)	C1—P1—C17—C18	−160.3 (2)
C6—C7—C8—C9	178.6 (3)	C13—P1—C17—C18	71.6 (3)
C7—C8—C9—C10	1.3 (6)	F3—C21—S1—O1	178.2 (3)
C8—C9—C10—C11	0.0 (5)	F2—C21—S1—O1	−61.4 (3)
C9—C10—C11—C12	−1.5 (5)	F1—C21—S1—O1	57.3 (3)
C10—C11—C12—C7	1.8 (4)	F3—C21—S1—O2	−60.5 (3)
C10—C11—C12—P1	−177.4 (2)	F2—C21—S1—O2	59.9 (3)
C8—C7—C12—C11	−0.5 (5)	F1—C21—S1—O2	178.6 (3)
C6—C7—C12—C11	179.8 (3)	F3—C21—S1—O3	58.8 (3)
C8—C7—C12—P1	178.9 (3)	F2—C21—S1—O3	179.2 (3)
C6—C7—C12—P1	−0.9 (3)	F1—C21—S1—O3	−62.1 (3)
C1—P1—C12—C11	179.1 (3)		

### Hydrogen-bond geometry (Å, º)

**Table:**

*D*—H···*A*	*D*—H	H···*A*	*D*···*A*	*D*—H···*A*
C2—H2···O1^i^	0.93	2.49	3.381 (4)	161
C19—H19*A*···O2^ii^	0.96	2.52	3.458 (4)	165
C11—H11···O3^iii^	0.93	2.7	3.601 (4)	162
C15—H15*B*···O3^iii^	0.96	2.69	3.470 (4)	138

Symmetry codes: (i) *x*+1/2, −*y*+1/2, −*z*+7/4; (ii) *y*, *x*, −*z*+2; (iii) *x*+1, *y*, *z*.

## References

[bb1] Allen, F. H. (2002). *Acta Cryst.* B**58**, 380–388. 10.1107/s0108768102003890 12037359

[bb2] Altomare, A., Burla, M. C., Camalli, M., Cascarano, G. L., Giacovazzo, C., Guagliardi, A., Moliterni, A. G. G., Polidori, G. & Spagna, R. (1999). *J. Appl. Cryst.* **32**, 115–119.

[bb3] Beletskaya, I. P. & Cheprakov, A. V. (2004). *J. Organomet. Chem.* **689**, 4055–4082.

[bb4] Brandenburg, K. & Putz, H. (2005). *DIAMOND* Crystal Impact GbR, Bonn, Germany.

[bb5] Bruker (1998). *SMART-NT* Bruker AXS Inc., Madison, Wisconsin, USA.

[bb6] Bruker (2008). *SADABS*, *SAINT-Plus* and *XPREP* Bruker AXS Inc., Madison, Wisconsin, USA.

[bb7] Farrugia, L. J. (2012). *J. Appl. Cryst.* **45**, 849–854.

[bb8] Flack, H. D. (1983). *Acta Cryst.* A**39**, 876–881.

[bb9] Herrman, W. A., Ofele, K., Preysing, D. & Schneider, S. K. (2003). *J. Organomet. Chem.* **687**, 229–248.

[bb10] Omondi, B., Shaw, M. L. & Holzapfel, C. W. (2011). *J. Organomet. Chem.* **696**, 3091–3096.

[bb11] Orlye, E. d’ & Jutland, A. (2005). *Tetrahedron*, **61**, 9670–9678.

[bb12] Sheldrick, G. M. (2008). *Acta Cryst.* A**64**, 112–122. 10.1107/S0108767307043930 18156677

[bb13] Williams, D. G. B., Shaw, M. L., Green, M. J. & Holzapfel, C. W. (2008). *Angew. Chem. Int. Ed.* **47**, 560–563. 10.1002/anie.200702889 18041799

